# Effect of Chemical Composition Variant and Oxygen Plasma Treatments on the Wettability of PLGA Thin Films, Synthesized by Direct Copolycondensation

**DOI:** 10.3390/polym10101132

**Published:** 2018-10-12

**Authors:** Muhammad Ayyoob, Young Jun Kim

**Affiliations:** Department of Chemical Engineering, Sungkyunkwan University, (16419) 2066 Seobu-Ro, Jangan-Gu, Suwon-Si, Gyeonggi-Do 16419, Korea; mayyoob@skku.edu

**Keywords:** direct esterification, PLGA, high molecular weight, copolymerization, wettability, hydrophobicity

## Abstract

The synthesis of high molecular weight poly (lactic-*co*-glycolic) acid (PLGA) copolymers via direct condensation copolymerization is itself a challenging task. Moreover, some of the characteristic properties of polylactide (PLA)-based biomaterials, such as brittleness, hydrophobicity, and longer degradation time, are not suitable for certain biomedical applications. However, such properties can be altered by the copolymerization of PLA with other biodegradable monomers, such as glycolic acid. A series of high molecular weight PLGAs were synthesized through the direct condensation copolymerization of lactic and glycolic acids, starting from 0 to 50 mol% of glycolic acid, and the wettability of its films was monitored as a function of the feed molar ratio. Copolymerization was performed in the presence of a bi-catalytic system using stannous chloride dihydrate and methanesulfonic acid (MSA). The viscosity average molecular weight of the resulting PLGA was in the range of 80k to 135k g/mol. The PLGA films were prepared using the solvent casting technique, and were treated with oxygen plasma for 2 min. The water contact angle of the PLGA films was determined before and after the oxygen plasma treatments, and it was observed that the wettability increased with an increase in the glycolic acid contents, however, the manifolds increased after 2 min of oxygen plasma treatments.

## 1. Introduction

Biodegradable polymers have recently been widely investigated as a result of increasing interest in biocompatibility and biosafety [1,2]. Linear poly(α-ester)s are the most interesting and widely investigated class of biodegradable polymers. Polylactides and polyglycolides as well as their copolymers are famous for their degradation profile, owing to labile ester linkages in their backbones, and the resulting products are safe, non-toxic, and biocompatible. Theoretically speaking, all classes of polyesters are susceptible to degradation, because of the reversibility of ester linkages, but for biomedical applications, only the polyesters with short aliphatic chains between these linkages are important [3].

Polyglycolic acid (PGA) is one of the first synthetic polymers, and it is studied for biomedical applications. PGA exhibits good tensile properties but poor thermal stability, as well as a very low solubility because of its high crystallinity (45–65%) [4,5,6,7]. Initially, the first biodegradable synthetic suture that was developed with PGA and was approved by the FDA (Food and Drug Administration USA) and marketed as DEXON^®^. It was observed during implantation, however, that the initial strength degradation time was short, as the tension was decreased by half within two weeks, as PGA is relatively hydrophilic; it can degrade completely within 60 to 90 days [5,8]. On the other hand, polylactide (PLA) is relatively more hydrophobic than PGA, because of the methyl group linked to the backbone of the polymer. A PLA device with high molecular weight will take almost five years to completely resorb [9]. PLA has been studied for biomedical and bone implants, such as a bone fixators [8]. Poly(l-lactic acid) (PLLA hereafter) also has been investigated as a scaffold in tissue engineering [8,10,11,12,13,14].

Poly(lactic-*co*-glycolic acid) (PLGA) is a copolymer of glycolic acid and lactic acid. The PLGA polymers with lactic acid contents higher than 25% are amorphous in nature. PLGA exhibits intermediate properties that mainly depend on the chemical compositions of both the constituents and their molecular weights. The copolymers usually show a higher rate of degradability than both the homopolymers glycolic and lactic acid [15,16]. PLGA 50:50 shows a very fast degradation rate that can take approximately two months, PLGA 75:25 can take up to five months, and PLGA 85:15 can take more than six months for complete degradation [17,18]. PLGA has been in use since the 1970s, but has earned increasing attention over the past two decades. The surgical sutures made with PLGA showed very promising results over natural and other synthetic sutures [19,20]. For PLGAs synthesized in different chemical compositions and for drug delivery, lower-molecular-weight PLGAs are preferred, while high molecular weight PLGAs are usually required for implants [21,22]. The synthesis of high molecular weight PLGAs is usually accomplished via ring-opening (ROP) using the cyclic diesters of the respective monomers [23]. The synthesis of a high molecular weight polymer through ROP is easier than condensation, whereas the preparation and purification of the cyclic diesters of both of the monomers (glycolide and lactide) is laborious and expensive. Hence, the overall ROP route of the PLGA synthesis is much more expensive compared with direct esterification. However, in polycondensation, it is difficult to achieve a high molecular weight of PLGA because of the poor thermal stability of the copolymer in the melt-state. There are two main reasons that limit the molecular weight of PLGA during direct esterification, namely: (1) the ineffective removal of condensate water during synthesis reaction and (2) the lowered thermal stability of the copolymer in the melt-state, which degrades by depolymerization and random chain scission at high temperatures.

PLGAs based biodegradable implants are widely used for several applications, such as Lactomer, a PLGA 70:30-based copolymer used to develop bone pins ligating clips [17,24,25,26]. Biodegradable implants are preferred because no post-repair surgery is required to remove the implant. Almost all of the practical applications of PGA, PLA, and PLGA involve bioresorption and biodegradation specific to the type of application; thus, it is very important that the biodegradation profile of the polymer is matched carefully to the lifespan required for the application type. There are several internal factors, such as the molecular weight of the polymer, monomer composition, tacticity, stereochemistry of the lactic acid units, the sequence of the monomeric units’ chain ends, and the dispersity index of the polymer, as well as external factors, such as the pH, temperature, enzymatic reactions, and iconic force [16,17,18,27,28]. Depending on the chemical composition and molecular weight, several commercially available biomedical products have been developed, including systems for meniscus and cartilage repair, guided tissue regeneration membranes for dentistry, sutures, tissue screws, drug delivery systems, bone fixators, micro and nano-spheres, implants, and tacks. PLGAs can also be used to develop and design the cardiovascular and urological stents for other applications, such as skin substitutes and skin fibroblast growth [29,30,31]. The wettability and chemical composition of the polymer that is used plays a significant role in protein entrapments [32]. The cell adhesion and protein absorption are highly effected by the wettability of the surface of the biomaterials, along with the surface charge, hydroxylation, and surface energy [33].

One of the factors affecting the degradation rates of the PLGA copolymers is the wettability of the implants or devices [34]. Despite the fact that PLA is less crystalline and more hydrophobic, while PGA is relatively more hydrophilic and highly crystalline, the crystallinity decreased rapidly in the copolymers of both. In order to adjust the properties of the polymers for a wider range of applications, PLGA copolymers with different chemical compositions are used. The changes in the chemical composition of copolymers can lead to changes in the hydration (surface wetting and swelling), hydrolysis (degradation), and amorphous. The manipulation of the characteristics of the PLGA-based biomaterials, such as their wettability, three-dimensional architectures, structural and mechanical integrity, biocompatibility, and their biodegradability, can be achieved using a feed ratio for both monomers. Previously, researchers have attempted to modify the PLA films in order to control the wettability and degradation rate using several techniques [35,36,37,38].

In the present work, a series of medium to high molecular weight PLGA copolymers were synthesized in the presence of a bi-catalysis system of methanesulfonic acid and tin chloride dihydrate, and were examined for the wettability of its thin films. A synthesis was performed at a relatively low temperature. Thin films were then cast using the solvent casting method and they were characterized for a water contact angle in order to determine the wettability of the copolymer films and the effect of the glycolic molar ratios on hydrophobicity of PLGA.

## 2. Materials and Methods

### 2.1. Materials

Glycolic acid, chloroform, tetrahydrofuran (THF), diphenylether, 3 Å molecular sieves and two catalysts (tin(II) chloride dihydrate and methanesulfonic acid), were purchased from Sigma Aldrich (Seoul, Korea) and were used as received. dl-lactic acid (predominantly containing l-isomer) in 85% to 90% aqueous solutions were acquired commercially from Alfa Aesar (Fisher Scientific Ltd., Incheon, Korea). 1,1,1,3,3,3-hexafluoro-2-propanol (HFIP) with a 99% purity was purchased from Fluorochem Ltd. (Derbyshire, UK), and was used as received. The 3 Å molecular sieves was activated using microwave oven (Samsung, Seoul, Korea) prior to use.

### 2.2. Synthesis of Copolymer

PLGA was synthesized with different monomer compositions of LA:GA, referred to as PLGA 10, PLGA 20, PLGA 30, PLGA 40, and PLGA 50, with lactic acid to glycolic acid ratios of 90:10, 80:20, 70:30, 60:40, and 50:50, respectively. In a typical synthesis procedure, a mixture of predetermined moles (depending upon molar ratio) of glycolic acid and dl-lactic acid were added to 250 mL three-neck round-bottom flasks, which were equipped with a mechanical stirrer (Luke GL^®^, Namyangju-si, Korea) and a vacuum line. The reaction mixture was dehydrated for four hours at 120 °C under a 200 Torr vacuum. The temperature was then raised to 140–160 °C, depending on the molar ratios after 30 min, and a catalyst (SnCl_2_·2H_2_O 0.30 g, Sigma Aldrich, Seoul, Korea) was added to the reaction mixture. Methanesulfonic acid (0.25 g at 2:1 MSA: SnCl_2_·2H_2_O molar ratio) and diphenylether were also added to the reaction mixture at this stage. The reaction mixture was dehydrated azeotropically over the pre-activated molecular sieves filled in “Soxhlet Extraction tube” (Luke GL^®^, Namyangju-si, Korea) under vacuum. After the predefined reaction time of 12 to several hours, the reaction was stopped by taking the flask out of the oil bath, the polymer was taken out of the three-neck flask, and it was then characterized without any further purification.

### 2.3. Characterization

The inherent and intrinsic viscosities of the synthesized PLGA were determined at a temperature of 30 °C using an unbeloved viscometer and a concentration of 0.5 dL/g in chloroform, unless otherwise mentioned. The viscosity average molecular weight was calculated, using Mark–Houwink constants of K and α, as mentioned in Equation (1). The differential scanning calorimetry (DSC) of PLGA was performed for the thermal properties, using a DSC Q20 TA Instrument (TA Instrument, New Castle, DE, USA), with a heating rate of 10 °C/min. An approximately 10 mg PLGA sample was prepared in an aluminum pan and temperature scanning was performed under a nitrogen flow with a temperature range of −20 to 200 °C. The glass transition temperatures were taken as the midpoint of the transition. The thermogravimetric analysis (TGA) was characterized on a Perkin Elmer TGA 7 thermal analyzer (Perkin Elmer, Billerica, MA, USA), with a heating rate of 10 °C/min under a nitrogen flow. An approximately 10 mg sample was charged into the sample pan, and characterization was performed under a nitrogen flow, with a heating rate of 10 °C and temperature scan ranging from 50 to 450 °C. ^1^H nuclear magnetic resonance (NMR) spectroscopy was performed in order to determine the molar ratios in the composition of the PLGA copolymers. Deuterated chloroform (CDCl_3_) was used as a solvent for the ^1^H NMR spectroscopy and was performed using a Bruker NMR 400 mhz (Bruker, Billerica, MA, USA).

### 2.4. Polymer Film Preparation

The polymer films were prepared via solution casting in chloroform with a 1 g/10 mL concentration. According to the predetermined thickness of the film, the PLGAs were dissolved in chloroform at room temperature. The solution was then poured into glass/Teflon Petri dishes (Cowie, New Castle, DE, USA) and kept at room temperature for three days. The films were then further dried under a vacuum at 30 °C for 24 h, and were characterized at room temperature for water contact angle measurements within 30 min of being removed from the oven (Lab House, Pocheon-si, Korea).

### 2.5. Oxygen Plasma Treatments

A PLA or PLGA film was cut in half. One half of the film was kept as a control sample, while the other half was treated on a reactive ion etching (RIE) plasma with an oxygen plasma at 30 W power with a gas flow rate of 5 cm/min for 2 min of treatment time.

### 2.6. Water Contact Angle Measurement of PLGA Copolymer Films

The water contact angle was measured using a water droplet imaging instrument containing a CMLN-13S2M-CS camera and a source of water droplets, made by Point Grey, Richmond, BC, Canada. A small portion of the films were cut off from each film sample and were pasted on the glass slides using a double-sided adhesive tape prior to measurement. At least five measurements of each PLGA copolymer film were taken in order to average out the water contact angle values. Image analyzer software (MeeSoft 1.33, Bangkok, Thailand) was used to calculate the water contact angle from the image that was captured after 0.80 s of contact time with water droplet.

### 2.7. Scanning Electron Microscopy of PLGA Films

In order to characterize the surface changes, the copolymer films were scanned under scanning electron microscopy using a JSM-7500F Field Emission Scanning Electron Microscope (JEOL, Tokyo, Japan) both prior to and after the oxygen plasma treatment. The SEM images of the surface of the polymer films were collected under 5.0 kV after a few nanometers of gold coating.

### 2.8. Measurements of Specific Optical Rotation

The specific optical rotation of the lactic acid monomer and all of polymers were also measured using a Perkin-Elmer PE-343 polarimeter (Perkin Elmer, Billerica, MA, USA). The lactic acid monomer was dissolved in water to prepare a solution of a 25 mg/dL concentration, and the degrees of the optical rotation were recorded from the polarimeter. Similarly, all of the polymers were dissolved in chloroform with a concentration of 1 g/dL, and the degrees of optical rotation were recorded. The sample pathlength was 1 dm and the standard sodium D-line at a 589 nm wavelength was used. The specific optical rotation was determined using following equation:

(1) $$\;{\left[\alpha \right]}_{D}^{20}=\frac{\alpha }{c\times l}\;$$

where ${\left[\alpha \right]}_{D}^{20}$ is the calculated specific optical rotation (deg dm^−1^ g^−1^ cm^3^) at 20 °C; α is the observed rotation in degrees from the polarimeter, measured using standard sodium D-line; c is the concentration of the polymer solution in g/mL; and l is the path length in dm. The specific optical rotation of the lactic acid monomer was −17.60 deg dm^−1^ g^−1^ cm^3^ in water.

## 3. Results and Discussion

### 3.1. Synthesis of PLA and PLGA Copolymers

**Scheme 1:**
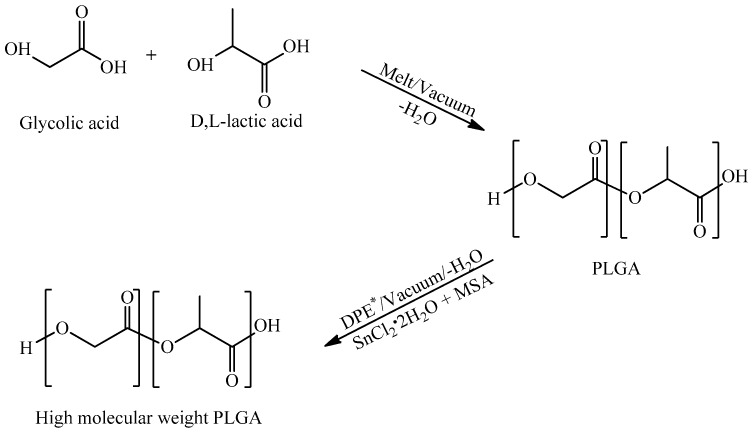
Synthesis scheme of high molecular weight poly(lactide-*co*-glycolide) (PLGA)-based biomaterials; * diphenylether.

Initially, a series of PLGA oligomers were synthesized with glycolic acid to a lactic acid molar feed ratio of 0/100 to 100/0. All of the polymers were synthesized at 180 °C for six hours under a vacuum, and the results are presented in Table 1. It was observed that the PLGAs with a higher proportion of glycolic acid were relatively less soluble in chloroform and took more time, and with 60% or more glycolic acid they were completely insoluble in chloroform. The copolymers from 0/100 to 50/50 were characterized for their inherent viscosities, and the results are nearly similar across all of the PLGA samples. All of the copolymer samples fell between 0.10 and 0.12, which indicates that all of the copolymers are low molecular weight oligomers. Later on, several experiments were performed in order to determine the optimal synthesis conditions, such as the temperature and vacuum. Two factors were kept under consideration, as follows: Firstly, when the synthesis of PLGA is at a high temperature, the resulting degradation of the polymeric chains is more likely than at lower synthesis temperatures, but at the same time, a longer reaction time is required. Secondly, the effective removal of water is important in order to overcome the reversible reaction balance towards the forward reaction. In order to synthesize high molecular weight PLGAs, diphenylether was usedas a solvent as shown in Scheme 1. Diphenylether and preactivated molecular sieves were used to remove the water effectively from the raction mixture.

**Figure 1:**
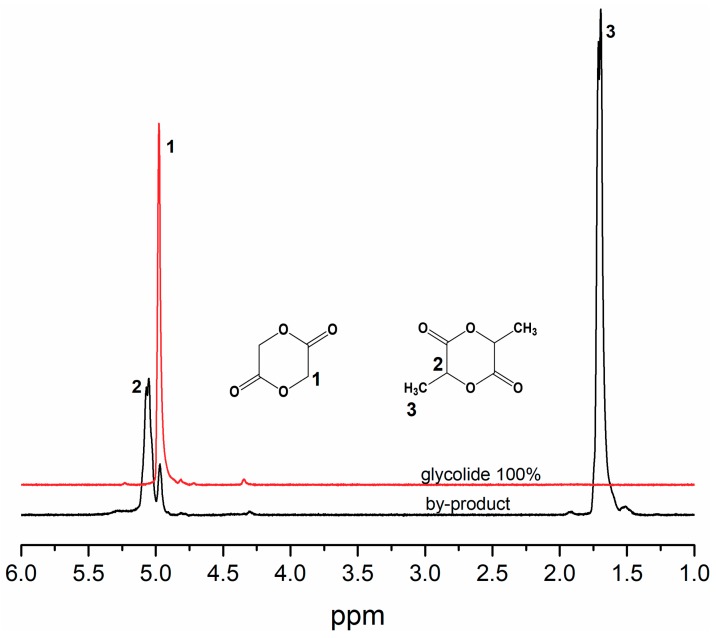
^1^H NMR analysis of by-products collected during synthesis of poly(lactide-*co*-glycolide) (PLGA) copolymers and pure glycolide.

It was noted that during the synthesis at high temperatures, such as 180 °C, or even higher under high vacuum, some of the by-products were omitted from the reaction mixture in the form of fumes. These vapors were condensed on the walls of the flask over the reaction mixture and also inside the vacuum tube, which crystalized upon cooling. These crystals were collected and analyzed by ^1^H NMR spectroscopy. The ^1^H NMR spectrum is shown in Figure 1. From the ^1^H NMR spectroscopy results, it was clear that this mixture was composed of glycolide and lactide, which was unsurprising, because it was obvious that in the presence of a tin catalyst at a high temperature, the depolymerization of copolymer chains can occur in the form of cyclization. Some of the monomers were removed from flask the under a high vacuum, which can reduce the molecular weight and yield as well. The direct esterification conditions were modified accordingly, because water removal is as important as the retention of cyclic diesters.

Keeping in mind that a higher molar ratio of glycolic acid in copolymer limits the solubility of polymers in a common organic solvent, a 0% to 50% molar ratio of glycolic acid was considered for the further synthesis of biomaterials for studying the wettability of PLGAs. Another series of copolymers were synthesized using diphenylether as solvent. While, the solvent was azeotropically dehydrated continuously under vacuum during whole synthesis time. The water evaporated along with solvent and absorbed in 3 Å molecular sieves when solvent passed through soxhlet tube contains molecular sieves. The direct esterification began at 120 °C and the temperature was raised to 140 °C/160 °C, the catalysts and solvent were added at this stage, and the reaction was then carried out at 140 °C or 160 °C, under a high vacuum (up to 10 to 20 Torr) in order to effectively remove any water and the results are presented in Table 2. A similar polycondensation reaction temperature for the synthesis of the PLGA copolymer was suggested as described previously [39]. The polycondensation was performed for 12 to several hours, and 0.61 to 0.86 dL/g intrinsic viscosities were obtained. The molecular weight was expressed in terms of the intrinsic viscosity, the PLGA polymers were characterized for the intrinsic viscosity, and the molecular weight was characterized using Kenley’s equation [40,41]:

(2) $$\left[\eta \right]=\left(1.07\times 10^{-4}\right)M_{v}^{0.761}\;$$

As the Mark–Houwik constants are specific to the polymer type, chemical composition, and solvent, it is quite difficult to determine the exact values of constants “K” and “α” for all of the chemical compositions of the PLGA copolymers with different molar feed ratios. However, both of the given references used different chemical compositions of PLGA in their studies, but the Mark–Houwik constants were the same in both of the studies [40,41]. Thus, we tried to determine the viscosity average molecular weight roughly using the above-mentioned constants from Kenley’s equation, and the results are presented in Table 2. For PLGA 50, which was synthesized for 24 h at 165 °C and 120 h at 140 °C, the intrinsic viscosity was determined to be 0.56 and 0.85 dL/g, respectively, in a THF at 30 °C, which represents the corresponding viscosity average molecular weights of 77k and 133k g/mol, respectively. The value of the viscosity average molecular weight (M_v_) theoretically lies between the weight average molecular weight (M_w_) and the number average molecular weight (M_n_), and is usually closer to the weight-average molecular weight, which may be approximately 10% to 20% less than the M_w_ [42]. However, Nieuwenhuis showed a trend in which the M_v_ was about 40% less than M_n_, and about 60% less than M_w_ [43]. In summary, we reached the point where the viscosity average molecular weight is supposed to be less than the weight average molecular weight, whether it is 20% or 60%. The PLGA prepared via direct esterification was in a range of a medium molecular weight (90k g/mol) to a high molecular weight (133k g/mol).

All of the homo- and co-polymers were characterized for optical properties, and they were presented as the specific rotation values. The specific rotation (α) values of the pure poly (l-lactic acid) and poly (d-lactic acid) are reported as −156 and +156 (degrees dm^−1^ g^−1^ cm^3^), respectively [44]. As mentioned earlier, lactic acid monomer used in this study predominantly contains l-lactic acid isomer with a specific optical rotation of −17.60 (deg dm^−1^ g^−1^ cm^3^). The lactic acid content in the copolymers were obviously poly (l-lactic acid) dominant as well, as it is clear from the specific optical rotation (α) results of the copolymers, shown in Table 2. A down trend is observed in (α) values with decrease in the feed mole% of lactic acid. PLA homopolymers shows a [α] value of 138 (deg dm^−1^ g^−1^ cm^3^), while on the other hand, PLGA 50 shows only 54 (deg dm^−1^ g^−1^ cm^3^). This down trend in the specific optical values is assumed because of the decrease in the overall concentration of the lactic acid in the copolymers, and not because of the racemization of lactic acid during the synthesis. The specific optical rotation values are considerably lower than those measured for the pure PLLA and PLLA-PGA random copolymers reported elsewhere. However, the decrease in the specific optical rotation values trend is almost the same [45].

### 3.2. Thermal Properties of PLA and PLGA Copolymers

**Figure 2:**
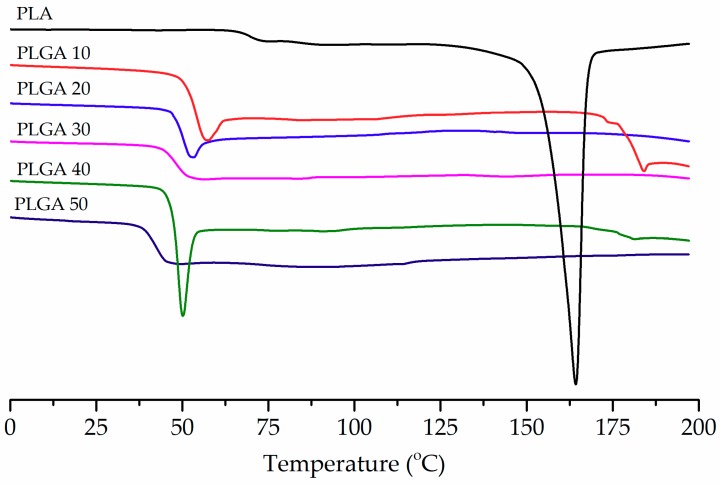
Differential scanning calorimetry (DSC) thermograms of polylactide (PLA) and PLGA, showing the thermal transition.

The copolymers were characterized for their thermal transition properties using differential scanning calorimetry (DSC), and the DSC thermograms are shown in Figure 2. From the DSC thermograms, the first, most important transition observed was the glass transition temperature. The glass transition temperature was 70 °C in the PLA scanning, and gradually decreased as the glycolic acid composition gradually increased. The T_g_ was observed at 70, 55, 51, 48, 48, and 44 °C from the DSC scanning of PLA and PLGA 10, 20, 30, 40, and 50, respectively. The T_g_ of the copolymers determined from the DSC thermograms is slightly lower than that of the T_g_ predicted using the Fox equation, which is calculated as 61, 57, 54, 52, and 49, respectively, for PLGA 10, 20, 30, 40, and 50. The Fox equation can be used to predict the T_g_ of the binary blends and copolymers [46].

The Fox equation can be used to predict the T_g_ of the binary blends and copolymers [46]. For the prediction of T_g_, using the Fox equation, the T_g_ of the PLA is assumed to be 65 °C, as reported in the literature, in the range of 54 to 65 °C [17,47,48], and the T_g_ of the PGA is considered as 40 °C, as shown in a range of 35 to 40 °C [1,4,49].

The second transition was only observed in the thermograms of the 100% PLA homopolymers at 162 °C, which were assigned as the melt transition. The PDLA homopolymers were believed to be the same as the amorphous polymer, but the melting peak shows some crystallization, which is attributed to the large amount of l-lactic acid in the monomer, as it is clear from the optical properties of the PLA and PLGA presented in Table 2. The PLLA segments in the homopolymers were crystallized and resulted in a melt transition during the differential scanning colorimetric analysis, but the melting temperature is considerably low compared with the pure PLLA with a similar molecular weight [50]. However, the melt transition of the PLA stereocomplexes of the homopolymers or the copolymers with glycolic acid mostly shifted towards a higher temperature range [28]. On the other hand, with the addition of the side chain-substituted PLA to the PLLA, a significant decrease in the melt transitions was observed [51]. In the presented results, any clear melt transition was not observed in any of the DSC thermograms of the PLGA copolymers, which shows that all of the copolymers are amorphous in nature. However, a relatively very weak melt transition was observed in PLGA 10, which is assumed to be because of the improper and defective crystallization of PLA because of the presence of a glycolic unit in the crystals. Almost similar results were observed when PLLA was copolymerized with PGA, except that the PLGA copolymer was only amorphous when the glycolic acid feed was over 30 mol%; however, the melting transition was decreased with an increase in the glycolic feed ratio [52].

**Figure 3:**
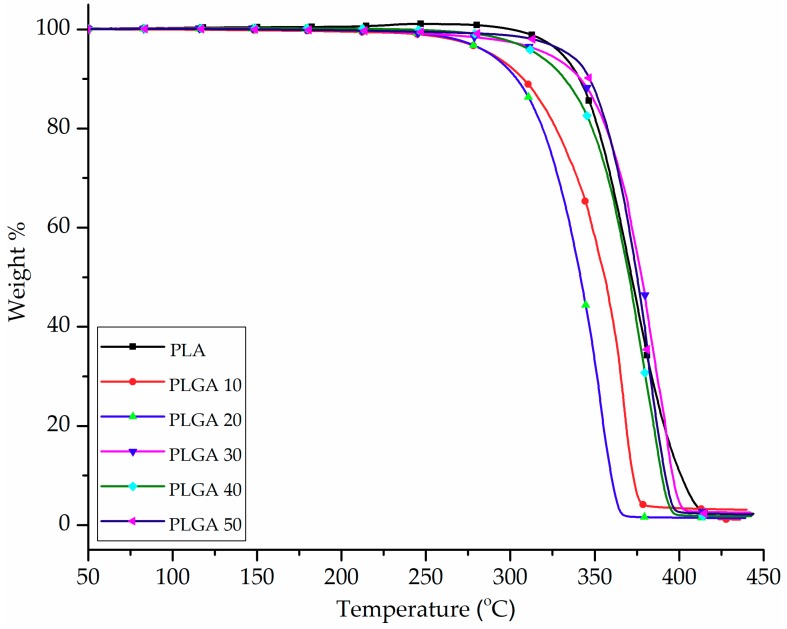
The thermogravimetric analysis (TGA) thermograms of the thermal stability of the PLA homo- and PLGA co-polymers with different chemical compositions.

The thermal degradation properties were characterized using a thermal gravimetric analysis. The thermal degradation of the PLGA copolymers was similar to that of the PLA homopolymers, as can be observed from the TGA thermograms in Figure 3. Except for the two polymer chemical compositions of PLGA 10 and 20, all of the other compositions were as thermally stable as the PLA homopolymers. PLGA 10 and 20 showed a lower thermal stability compared with the PLA homopolymers and other chemical composition, which is supposedly mainly because of the lower molecular weight, as the results show in Table 2. The thermal degradation window of PLGA 10 and 20 was in between 310 and 380 °C, while other compositions of PLGA mainly degraded from 360 to 400 °C. Thus, overall, no significant effect of chemical composition on the thermal stability of the copolymers was observed. The thermal properties are similar to the previously reported PLGA copolymers [53].

**Figure 4:**
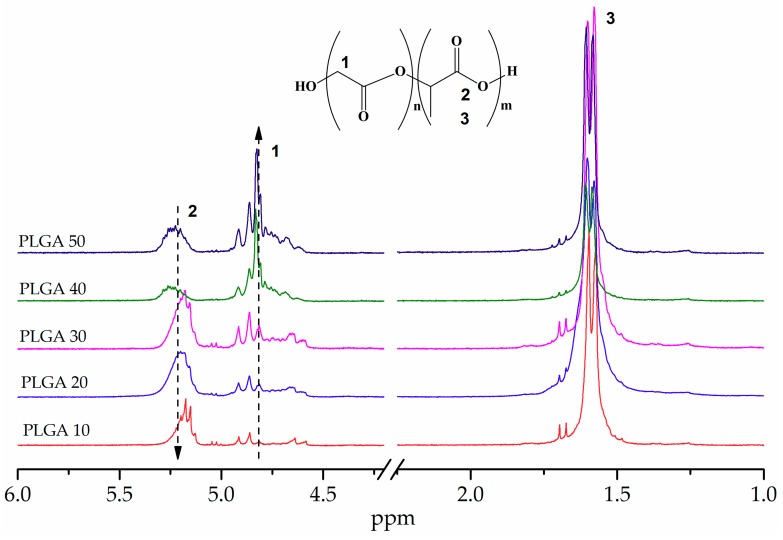
^1^H NMR spectrograms, an increase in the peak intensity of the chemical shift of glycolydyl unit at 4.8 ppm.

The chemical compositions of PLGA in the copolymers were determined using ^1^H NMR spectroscopy. The molar ratio of the glycolic acid and lactic acid in the feed and the ratio in the PLGA, calculated by the ^1^H NMR spectra (Figure 4), is tabulated in Table 2. The ratio of glycolic acid in the copolymer was determined based on the integrals of the area of the proton signals in the ^1^H NMR spectra at 1.66 to 1.68, attributed to the –CH_3_ protons of the lactic acid contents at 4.7 to 5.03 ppm, was assigned to the –CH_2_ protons of glycolic acid, and at 5.2 to 5.5 ppm, were attributed to the –CH protons of the LA (lactic acid) units.

The equations given below were used to calculate the molar percentage of the glycolic acid component in the copolymer [54]:

(3) $$\;P_{GA}=\;\frac{I_{G}}{\left(2I_{L}+I_{G}\right)}\;$$

The percentages of glycolic acid determined from ^1^H NMR in the copolymers were 16.6, 22.7, 31.8, 47.9, and 55.5% for PLGA 10, 20, 30, 40, and 50, respectively. As is clear from the ^1^H NMR spectroscopy, the glycolic acid contents were higher in the copolymer than the feed percentages in the reaction mixture. The results of the glycolic acid and lactic acid ratios in the copolymers are not surprising, as these values are in close accordance with the reported results of random PLGA copolymers [54]. As shown in Figure 4, notable changes were observed in the peak intensities from the ^1^H NMR spectra at 4.4 and 4.5, the region assigned to the glycolydyl units. The intensities of the glycolydyl units increased significantly as the percentage of glycolic acid increased in the reaction mixture. There is no evidence of the cyclization of depolymerization forming cyclic diesters during the synthesis of PLGA, as no glycolide peak was observed at 5.08 ppm, nor were peaks for any the dimers observed in the spectral region of 4.28 to 4.78 ppm. The peaks observed at 4.70 and 4.94 ppm can be assumed to be the spinning sideband or carbon satellites of glycolide [55].

### 3.3. Surface Analysis and Wettability Results of Polymer Films

**Figure 5:**
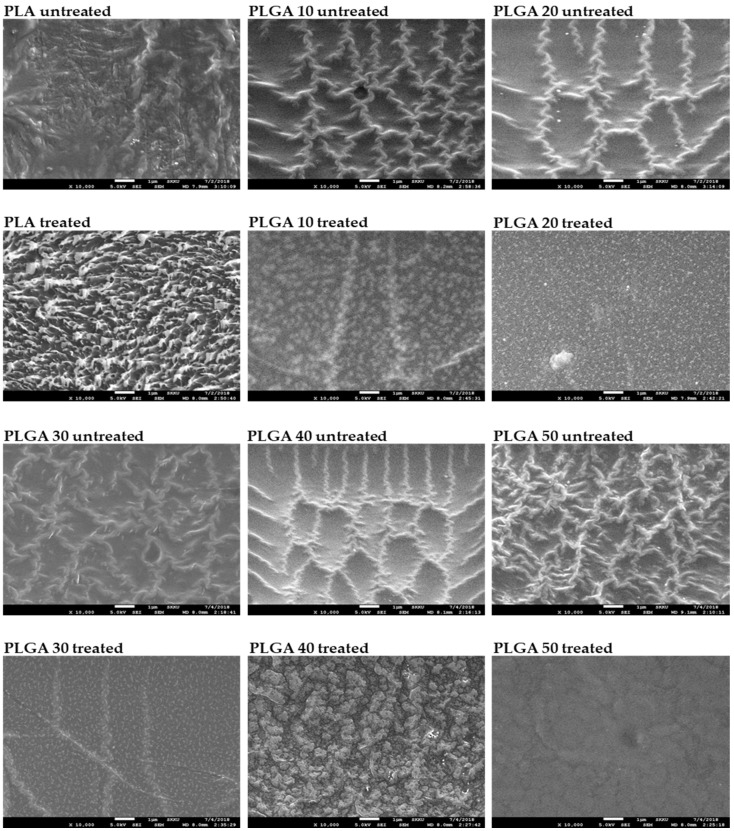
SEM images of polymer films, prior to and after oxygen plasma treatments.

**Figure 6:**
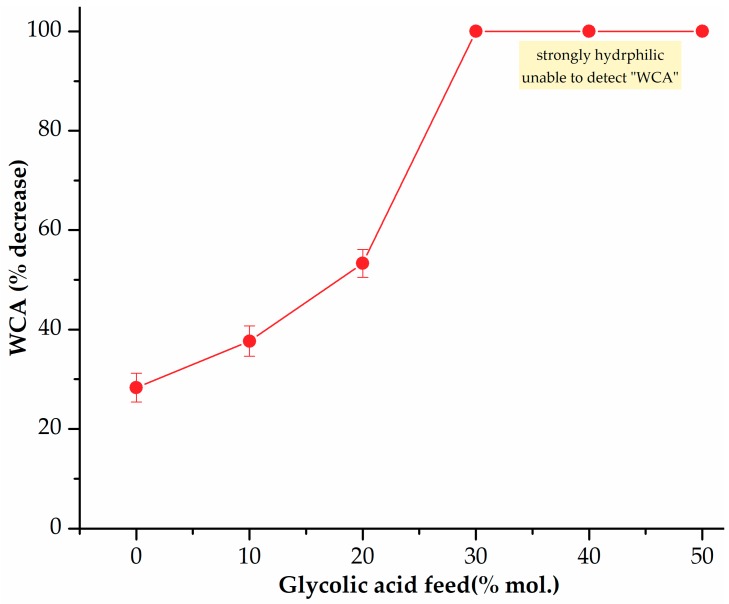
After 2 min of oxygen plasma treatment, the % decrease in the water contact angle (WCA). A higher percentage decrease is in the WCA of the films with higher glycolic acid contents.

**Figure 7:**
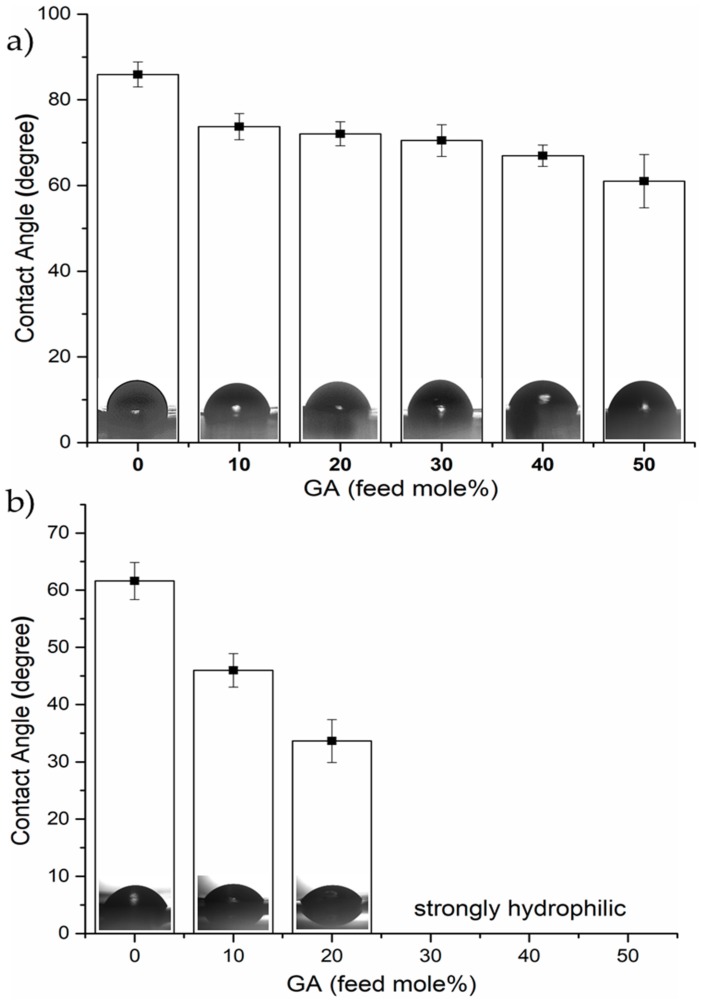
Water contact angle characterization of homo- and co-polymer films prior to and after plasma treatments.

In order to investigate the surface changes of the PLA and PLGA films, SEM was used; corresponding SEM images are shown in Figure 5. A thread-like structure can be seen in PLA homo- and co-polymer films as the glycolic acid in the copolymer increased from 0 to 50%. This channel-like structure in the SEM images might be as the result of some sort of phase separation of the glycolydyl units from the lactidyl units, keeping out the methyl unit of PLA. Furthermore, a clear change in terms of dark and light contrast can be observed on the surface of the oxygen plasma treated films. It was assumed that these white spots appeared as a result of the oxygen ion bombardment during the plasma treatment, which resulted in the surface roughness of polymer films. The wettability of the polymer films was improved because of the aforementioned surface roughness. The polymer films were characterized for WCA, prior to and after the oxygen plasma treatments. The water contact angle (WCA) values after measurement are presented in Table 2, with graphical presentations in Figure 6 and Figure 7.

The WCA decreased significantly from 86° to 73°, with the addition of a low quantity of glycolic acid, even just 10 mol%. The contact angle decreased gradually with an increase of glycolic acid contents in PLGA copolymers. We know that the methyl group in the polylactic acid backbone reduces the hydrophilicity and contributes to its role making polymers hydrophobic. With the addition of glycolic acid in random copolymers of lactic acid and glycolic acid, the polymer chain concerted into lactic and glycolic segments, reducing the hydrophobicity of the copolymer and/or increasing the hydrophilicity. However, an increased hydrophilicity means an increased water uptake, which can ultimately result in a higher rate of degradation. The water sorption and degradation can be influenced significantly by the wettability of the polymer surface, mainly because of the catalytic effect of the by-product an produced during hydrolysis of polymer, if any, on the surface of polymer [36,37]. Thus, the copolymerization of lactic acid and glycolic acid can be used to modify the wettability of the PLGA copolymer, and consequently cause the adjustable degradation rate of biomaterials.

Hypothetically speaking, the wettability of almost of every kind of polymer or polymer blend can be adjusted using plasma treatments [56]. On the other hand, when the oxygen plasma was applied for only 2 min, the results were quite impressive and somehow unpredictable. The wettability was increased sharply and a trend was observed in the increasing wettability, starting from 100% PLA to PLGA 50. A WCA of 100% PLA was dropped considerably, from 86° to 61°, which is about a 30% decrease in WCA as shown in Figure 6. Similar results were observed for PLGA 10 and PLGA 20, where 37% and 54% drops in the WCA were observed, respectively. The wettability of the copolymers films of PLGA 30, 40, and 50, were too high to calculate the WCA, as shown in Figure 7. The water droplet spread was too fast and it was not possible to take an image. Similar results were reported by Kiss et al., except that the PLA and PLGA films were modified by adding Pluronic PE6800 [38]. The results presented here after plasma treatments are quite similar to that of the PLA/PCL (polycaprolactone) sponge plasma treatment for the tissue engineering biomaterial [56].

Moreover, as it is clear from the SEM images, because of the bombardment of the oxygen ion, the topology of the polymer film surface is largely changed, which affects the hydrophilicity of copolymers. However, there are possibilities of more hydrophilic oxygen atoms on the surface films, as a result of the oxygen plasma. Those hydrophilic interactions can result in a more spread out water droplet, lowering the WCA. From such an observation, a conclusion can be drawn that, with the control of the copolymerization feed ratio and the oxygen plasma treatment, the wettability of the PLA- and PLGA-based biomaterials can largely be controlled both effectively and efficiently.

## 4. Conclusions

A high molecular weight PLGA was synthesized via direct esterification under mild conditions. At its peak, a 133k g/mol molecular weight was achieved in 48 h at 140 °C. It has been confirmed from the byproducts that a polymer chain degradation and cyclization can occur during synthesis at a relatively higher temperature (180 °C or over) and under a high vacuum (5–10 Torr). The higher molar ratio in the copolymers compared with the feed ratio is attributed to the higher reactivity of glycolic acid. The contact angle analysis shows an improvement in the wettability of the PLGA copolymers, with increasing mol% of glycolic acid contents. The wettability increased by manifolds after only 2 min treatment of oxygen plasma. The water contact angle was decreased from 86° to 60° in the case of the PLA homopolymers, while in the case of PLGA 20, it decreased from 72° to 33°. The wettability of PLGA 50 was too high, thus the water contact angle measurements were not possible after the plasma treatment. The wettability of the PLGA-based biomaterials, demonstrated as a function of the feed molar ratios of the monomers, and a enhanced wettability was observed after the plasma treatments. 

## Figures and Tables

**Table 1:** Synthesis of polylactic-*co*-glycolic acid oligomers with different chemical compositions.

	Lactic Acid/Glycolic Acid (mol. ratio)	η * (30 °C/CHCl_3_)	Solubility (chloroform)
PLA 100/0	100/0	0.11	++++
PLGA 99/01	99/01	0.12	++++
PLGA 98/02	98/02	0.11	++++
PLGA 95/05	95/05	0.11	++++
PLGA 90/10	90/10	0.11	++++
PLGA 80/20	80/20	0.12	++++
PLGA 65/35	65/35	0.12	+++
PLGA 50/50	50/50	0.11	+++
PLGA 35/65	35/65	Not soluble	++
PLGA 20/80	20/80	Not soluble	+
PLGA 10/90	10/90	Not soluble	−
PLGA 05/95	05/95	Not soluble	−
PLGA 02/98	02/98	Not soluble	−
PLGA 01/99	01/99	Not soluble	−
PLGA 0/100	0/100	Not soluble	−

* Inherent viscosity. PLA—polylactide; PLGA—poly(lactide-*co*-glycolide).

**Table 2:** Synthesis and results of PLA homopolymers and PLGA copolymers.

Sample Code	Yield (%)	${\left[\alpha \right]}_{D}^{20}\;(\deg\;{\mathrm{dm}}^{-1}\;g^{-1}\;{\mathrm{cm}}^{3}){\;}^{b}$	[η] * (dL/g)	M_v_ (g/mol.)	T_g_ (°C)	WCA ^a^ (degrees)		Film Thickness (μm)	Glycolic Acid Contents (mol.%)	
						Untreated	Treated		In the Feed	In the Copolymer
PLA	91.3	−138	0.83	130,000	70	85.9	61.6	130	0	0
PLGA 10	82.6	−135	0.65	94,000	55	73.3	46.0	120	10	16.7
PLGA 20	88.4	−124	0.62	88,000	51	72.0	33.6	130	20	22.1
PLGA 30	89.2	−108	0.81	125,000	48	69.0	N/D	120	30	31.9
PLGA 40	83.8	−85	0.78	120,000	48	66.8	N/D	150	40	47.9
PLGA 50	84.7	−54	0.75	113,000	44	61.0	N/D	170	50	55.5
PLGA 50	81.6	N/D	0.86	133,000	46	62.1	N/D	160	50	61.3

^a^: Water contact angle; ^b^: the unit of specific optical rotation is $\frac{\mathrm{degrees}}{\mathrm{dL}\times g/{\mathrm{cm}}^{3}}$; *: intrinsic viscosity measured at 30 °C, using CHCl_3_ as solvent; N/D: specific optical rotation was not determined.
